# Diurnal circadian clock gene expression is altered in models of genetic and acquired epilepsy

**DOI:** 10.1002/epi4.12841

**Published:** 2023-10-16

**Authors:** Glenn R. Yamakawa, Meshwa Patel, Runxuan Lin, Terence J. O'Brien, Richelle Mychasiuk, Pablo M. Casillas‐Espinosa

**Affiliations:** ^1^ Department of Neuroscience, Central Clinical School Monash University Melbourne Victoria Australia; ^2^ Department of Neurology The Alfred Hospital Melbourne Victoria Australia

**Keywords:** animal model, *bmal1*, circadian, *cry1*, GAERS, *per1*, post‐SE, seizure, sleep

## Abstract

**Objectives:**

Growing evidence demonstrates a relationship between epilepsy and the circadian system. However, relatively little is known about circadian function in disease states, such as epilepsy. This study aimed to characterize brain and peripheral core circadian clock gene expression in rat models of genetic and acquired epilepsy.

**Methods:**

For the Genetic Absence Epilepsy Rats from Strasbourg (GAERS) study, we used 40 GAERS and 40 non‐epileptic control (NEC) rats. For the kainic acid status epilepticus (KASE) study, we used 40 KASE and 40 sham rats. Rats were housed in a 7 am:7 pm light–dark cycle. Hypothalamus, hippocampus, liver, and small intestine samples were collected every 3 h throughout the light period. We then assessed core diurnal clock gene expression of *per1*, *cry1*, *clock*, and *bmal1*.

**Results:**

In the GAERS rats, all tissues exhibited significant changes in clock gene expression (*P* < 0.05) when compared to NEC. In the KASE rats, there were fewer effects of the epileptic condition in the hypothalamus, hippocampus, or small intestine (*P* > 0.05) compared with shams.

**Significance:**

These results indicate marked diurnal disruption to core circadian clock gene expression in rats with both generalized and focal chronic epilepsy. This could contribute to epileptic symptomology and implicate the circadian system as a viable target for future treatments.

**Key Points**

- Genetic Absence Epilepsy Rats from Strasbourg showed dysregulation of all circadian clock genes, *per1*, *cry1*, *clock*, and *bmal1* in brain and peripheral tissue and had an epilepsy by time interaction.
- Kainic acid status epilepticus rats had increased *Cry1*, *Clock*, and *Bmal1* expression in the hypothalamus and liver, and *Cry1* and *Clock* in the hippocampus compared with shams.
- Altered clock gene expression is a novel marker in the pathogenesis of epilepsy.

## 1 INTRODUCTION

Epilepsy is a common and serious chronic brain condition comprising heterogenous syndromes and unprovoked, recurrent seizures that affect 7.6 per 1000 people across the globe.^1^ Drug‐resistant epilepsy (ie, when antiseizure medications [ASM] cannot control seizures) accounts for one‐third of the affected population.^2^ Nearly 75% of epilepsy cases emerge in childhood and is a lifelong condition.^3^ In working‐age epilepsy patients, it was estimated that 146 000 years of total adjusted life was lost, in addition to $22.1 USD billion gross domestic product.^4^ The high prevalence rates, mortality, and costs to society emphasize the urgency required to develop targeted therapeutics for drug‐resistant epilepsy.

For optimal survival, most living organisms, including animals, plants, and microbes anticipate environmental changes, such as food availability, temperature, and light.^5^, ^6^ To adjust to cyclic environmental changes, mammals exhibit rhythms in physiology and behavior, including sleep–wake cycles and oscillations in metabolic, cardiovascular, endocrine, immune, and neurological functions.^7^ These are known as circadian rhythms as they are entrained to the solar day. The hypothalamic suprachiasmatic nucleus (SCN) houses the central clock, which is the primary regulator of circadian rhythms.^8^ The molecular basis of these circadian rhythms are generated by the core circadian clock genes; period (*Per 1*, *2*, *3*), cryptochrome (*Cry 1*, *2*), circadian output cycles kaput (*clock*), and brain and muscle ARNT‐Like 1 (*bmal* 1).^7^ There are additional accessory stabilizing loops involving *casein kinase* 1 (Ck1) and *rev‐erb‐alpha* (Nr1d1).^9^, ^10^ When c*lock* and *bmal1* are expressed, they heterodimerize before translocating back to the nucleus where they stimulate the expression of *Per 1*, *2*, *3*, and *Cry 1*, *2*.^11^ *Per* and *Cry* heterodimerize before they translocate back to the nucleus to suppress the expression of *Clock*/*Bmal1*, thereby halting their own expression in a negative feedback loop that takes approximately 24 h to complete.^12^

There is a bidirectional relationship between circadian rhythms and epilepsy, where seizures can alter circadian rhythms, while seizures also follow a circadian pattern. However, while a significant number of seizures display a circadian pattern, exceptions exist.^13^, ^14^ Inadequate sleep can increase drowsiness during the day and cognitive problems, leading to worse seizure control.^15^ Epileptic seizures have been shown to produce changes in sleep activity and influence circadian rhythms due to the epileptogenic pathology.^16^ Altered sleep patterns have been observed in post‐status epilepticus (SE) rat models of chronic temporal lobe epilepsy (TLE), after 15 weeks from the induction of epilepsy.^17^ Induction of TLE in the mice pilocarpine model was also found to suppress circadian dynamics in EEG, but as rhythms were restored, spontaneous seizures became clustered in a circadian manner.^18^

The Genetic Absence Epilepsy Rats from Strasbourg (GAERS) is a prominent model that resembles the pharmacological features, electro‐clinical behavior, and pathophysiology of human idiopathic generalized epilepsy with absence epilepsy.^19^, ^20^ The GAERS were selectively inbred to develop absence seizures, while their counterparts the non‐epileptic control (NEC) rats, were inbred from the same original Wistar strains as GAERS but selectively inbred not to express seizures.^19^ Much like the human condition, seizures in GAERS are characterized by spike–wave discharges (SWD) on the EEG. Additionally, GAERS manifest anxiety and depressive‐like behavior, cross‐modal recognition memory, which represent neuropsychiatric comorbidities commonly present in the human condition.^21^, ^22^, ^23^ Furthermore, studies over the past few decades have revealed that GAERS seizures have a similar ASM therapy response to those individuals suffering from absence epilepsy.

One of the most widely used models to study chronic drug‐resistant TLE is the kainic acid (KA) induced post‐SE (KASE) model. Kainic acid, a glutamate agonist, is used to induce SE, which acts as the precipitating brain insult, that triggers the epileptogenic process. After a 2–4 week latent period, animals develop TLE, which gradually becomes resistant to drug treatment, at the time in which animals develop comorbidities, which are analogous to human drug‐resistant TLE.^24^

This study aimed to assess core circadian clock gene diurnal expression in different epilepsy types, using the GAERS model of IGE, and the KASE rat model of TLE. We assessed diurnal expression of core clock genes *Per1*, *Cry1*, *Clock*, and *Bmal1* expression across the brain (hypothalamus and hippocampus) and periphery (liver and small intestine) to understand circadian dysfunction and desynchronization across the nervous system. We hypothesized that diurnal clock gene rhythms would be disrupted in both chronic epilepsy models, in a tissue and time‐dependent manner.

## 2 MATERIALS AND METHODS

### 2.1 Experimental design

Animals were housed in pairs, on a 12:12 light–dark cycle (lights on at 0700) in a temperature (22 ± 2°C) and humidity‐controlled environment for the duration of the study. Food (standard rat chow) and water were available ad libitum. All rats were entrained to the light–dark cycle for at least a week prior to tissue collection. Efforts were made to limit the number of animals used and to minimize suffering. Experimental procedures were approved by the Alfred Research Alliance Animal Ethics Committee (ARA‐AEC ethics numbers E/2034/2020/M and E/1979/2019/M) and adhered to the Australian Code for the care and use of animals for scientific purposes.

### 2.2 Circadian gene expression

#### 2.2.1 GAERS study

For the GAERS cohort, (*n* = 40) NEC and (*n* = 40) GAERS aged 11 weeks were obtained from our colony located at the Monash Animal Research Platform (Monash University).

#### 2.2.2 Kainic acid‐induced status epilepticus study

For the KASE cohort studies, male Wistar rats (*n* = 80) rats were purchased from the Animal Resource Centre (Perth, Australia). As previously described, 11‐week‐old male Wistar rats (*n* = 40) underwent a modified low‐dose KA intraperitoneal administration protocol.^24^, ^25^ Age‐matched (sham, *n* = 40) controls were handled the same way during the same experimental session as the KASE rats did not receive KA but receive intraperitoneal saline injection (1 mL/kg) instead. Following the initial injection of KA (7.5 mg/kg ip), the rats were monitored for 45 min for behavioral seizures by an experimenter blinded to the drug administration (KA or saline) using the Racine scale. If continuous class IV‐V seizures indicative of the emergence of SE were not observed, a second dose of KA 5 mg/kg was administered. Subcutaneous sterile physiological saline injections were administered to prevent dehydration of the animals. Further KA doses of 2.5 mg/kg every 30–45 min until SE was observed for a maximum of 20 mg/kg. The first KA injection occurred at 8 am, and the following injections were administered between 8 am and 11 am. Diazepam (5 mg/kg) was used to stop the SE after 4 h. Status epilepticus was stopped between 2 pm and 3 pm.^25^

#### 2.2.3 Tissue collection

GAERS (*n* = 40) and NEC (*n* = 40), aged 20 weeks, KASE (*n* = 40) and shams (*n* = 40), 9 weeks post‐SE (20 weeks of age) induction, were randomly assigned to a tissue collection time, using a random number generator algorithm. Rats, GAERS (*n* = 8) and NEC (*n* = 8), KASE (*n* = 8) and shams (*n* = 8) per timepoint were euthanized every 3 h throughout the day at Zeitgeber time (ZT) 0, 3, 6, 9, and 12 with ZT0 being defined as lights on and ZT12 lights off. Rats were placed in an induction chamber and deeply anesthetized with isoflurane (Primal Enterprises LTD) until they had lost the toe pinch reflex. Then, animals were removed and euthanized. The hypothalamus was dissected posterior to the optic chiasm since the SCN has been found to run in antiphase to other hypothalamic regions.^26^, ^27^ The whole hippocampus was dissected bilaterally. The brain regions were selected as they are critical for circadian rhythm regulation.^28^ A 1‐cm portion of the liver's left lobule was removed, and a 1‐cm portion of the proximal jejunum was removed from the small intestine. All the tissue was flash frozen on dry ice and then stored at −80°C.

#### 2.2.4 Quantitative real‐time polymerase chain reaction

RNA was extracted from tissue using RNeasy kits following the manufacturer's instructions (Qiagen). RNA was reverse transcribed to cDNA using qScript Ultra SuperMix following the manufacturer's instructions (QuantaBio). Quantitative real‐time polymerase chain reaction (qRT‐PCR) was run in duplicate on 384‐well plates using the Qiagility system (Qiagen). The final concentration in each well was 20 ng of cDNA template, 1X SYBR green fastmix ROX, and 0.5 μM forward and reverse primers. Standards curves were generated and compared against background expression of housekeeping genes.^29^ Primers used are displayed in Table 1. qRT‐PCR was run with the Quantstudio 7 Flex Real Time PCR system (Thermo Fisher Scientific). PCR data were extracted and analyzed using the 2^−∆∆Ct^ method to calculate relative fold gene expression and quantified against the housekeeping genes.^30^ In Table 1, the primers display the primer sequences used to conduct qRT‐PCR. All primers were purchased from Integrated DNA Technologies. Forward: 5′‐3’ Reverse: 3′‐5′. *per1*: Period1, *cry1*: Cryptochrome1 CLOCK: Circadian Locomotor Output Cycles Kaput BMAL1: Brain and Muscle Arnt‐Like protein Ywhaz: Tyrosine 3‐monooxygenase CycA: Cyclophilin A.

**TABLE 1:** Primers.

Primers	Sequence
*Per1*	
Forward	CTG CAA CAT TCC TAA CAC AAC C
Reverse	GAA GCT ACA CTG ACT GGT GAC G
*Cry1*	
Forward	TTC CAG ACA TCA TTG TTT GAC C
Reverse	TGA ACA AGA AGG GAG ACA AAG G
*Clock*	
Forward	CAG AAG TTA GGG CTG AAA GAC G
Reverse	GAG GAC TTT CTT GAG CTT CTG G
*Bmal1*	
Forward	TGC CAC CAA TCC ATA CAC AG
Reverse	TTC CCT CGG TCA CAT CCT AC
*Ywhaz*	
Forward	TTG AGC AGA AGA CGG AAG GT
Reverse	GAA GCA TTG GGG ATC AAG AA
*Cyca*	
Forward	AGC ACT GGG GAG AAA GGA TT
Reverse	AGC CAC TCA GTC TTG GCA GT

### 2.3 Statistical analyses

All data were analyzed using IBM SPSS 25 for Windows. Two‐way ANOVAs were used to determine statistical significance with condition (NEC/GAERS or control/KASE) and time (circadian time 0, 3, 6, 9, 12) as factors. *P* < 0.05 being set as the threshold for significance. GraphPad Prism 8 (version 8.3.1 for windows, GraphPad software) was used to graph the results that are displayed as means ± standard errors.

## 3 RESULTS

### 3.1 GAERS diurnal circadian gene expression studies

#### 3.1.1 Hypothalamus

**FIGURE 1:**
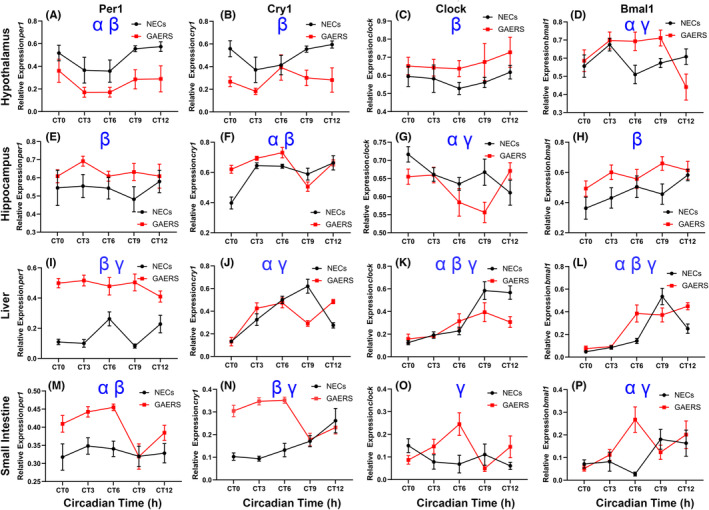
Gene expression for non‐epileptic control (NECs) and Genetic Absence Epilepsy Rats from Strasbourg (GAERS). The hypothalamus is displayed in panels (A–D), the hippocampus is displayed in panels (E–H), the liver is displayed in panels (I–L), and small intestine in panels (M–P). *α* represents a main effect for time; *β* represents a main effect for epilepsy. *γ* represents a condition by time interaction. X‐axis is displayed in circadian time hours where CT0 is defined as the start of the day. The Y‐axis displays the expression of each gene of interest relative to housekeeping genes. GAERS (*n* = 8) and NEC (*n* = 8) per CT.

In the hypothalamus, *Per1* expression showed a main effect of epilepsy, *F*_(1, 73)_ = 18.38; *P* < 0.01, and time *F*_(4, 73)_ = 2.55; *P* < 0.05. For *cry1*, there was a main effect of epilepsy, *F*_(1, 76)_ = 19.41; *P* < 0.001. For *Clock*, there was a main effect of epilepsy, *F*_(1, 76)_ = 5.88; *P* < 0.05. *Bmal1* showed a main effect for time, *F*_(4, 75)_ = 4.28; *P* < 0.01, and a significant condition by time interaction, *F*_(4, 75)_ = 3.45; *P* < 0.05. Results for hypothalamic gene expression are displayed in (Figure 1A–D).

#### 3.1.2 Hippocampus

*Per1* showed a significant main effect for epilepsy, *F*_(1, 79)_ = 5.74; *P* < 0.05. *Cry1* showed a significant main effect for epilepsy, *F*_(1, 79)_ = 8.34; *P* < 0.01, time *F*_(4, 79)_ = 14.09; *P* < 0.01, and a epilepsy by time interaction, *F*_(4, 79)_ = 6.96; *P* < 0.001. *Clock* showed a significant main effect for time, *F*_(4, 79)_ = 2.947; *P* < 0.05, and a significant epilepsy by time interaction, *F*_(4, 79)_ = 2.956; *P* < 0.05. *Bmal1* showed a significant main effect for epilepsy, *F*_(1, 79)_ = 9.744; *P* < 0.01 (Figure 1E–H).

#### 3.1.3 Liver

In the liver, there was a main effect for condition for *Per1*, *F*_(1, 78)_ = 157.798; *P* < 0.01, and a significant epilepsy by time interaction, *F*_(4, 78)_ = 4.076; *P* < 0.01. *Cry1* had a main effect for time, *F*_(4, 79)_ = 26.395; *P* < 0.01, and an epilepsy by time interaction, *F*_(4, 79)_ = 13.793; *P* < 0.01. *Clock* showed a main effect for epilepsy, *F*_(1, 79)_; *P* < 0.04, time *F*_(4, 79)_ = 17.562; *P* < 0.01, and a epilepsy by time interaction, *F*_(4, 79)_ = 4.205; *P* < 0.01. *Bmal1* showed a main effect for epilepsy, *F*_(1, 79)_ = 5.343; *P* < 0.05, time, *F*_(4, 79)_ = 30.864; *P* < 0.01, and an epilepsy by time interaction, *F*_(4, 79)_ = 7.206; *P* < 0.01 (Figure 1I–L).

#### 3.1.4 Small intestine

In the small intestines, there was a main effect for epilepsy in *Per1* expression *F*_(1, 79)_ = 20.831; *P* < 0.01, and a main effect of time, *F*_(4, 79)_ = 3.385; *P* < 0.05. *Cry1* expression showed a main effect for epilepsy, *F*_(1, 79)_ = 58.325; *P* < 0.01, and a significant epilepsy by time interaction, *F*_(4, 79)_ = 11.837; *P* < 0.01. *Clock* showed significant epilepsy by time interaction *F*_(4, 79)_ = 4.303; *P* < 0.01. *Bmal1* displayed a significant main effect of time, *F*_(4, 77)_ = 2.766; *P* < 0.05, and a significant epilepsy by time interaction, *F*_(4, 77)_ = 3.893; *P* < 0.01 (Figure 1M–P).

### 3.2 KASE diurnal circadian gene expression studies

#### 3.2.1 Hypothalamus

**FIGURE 2:**
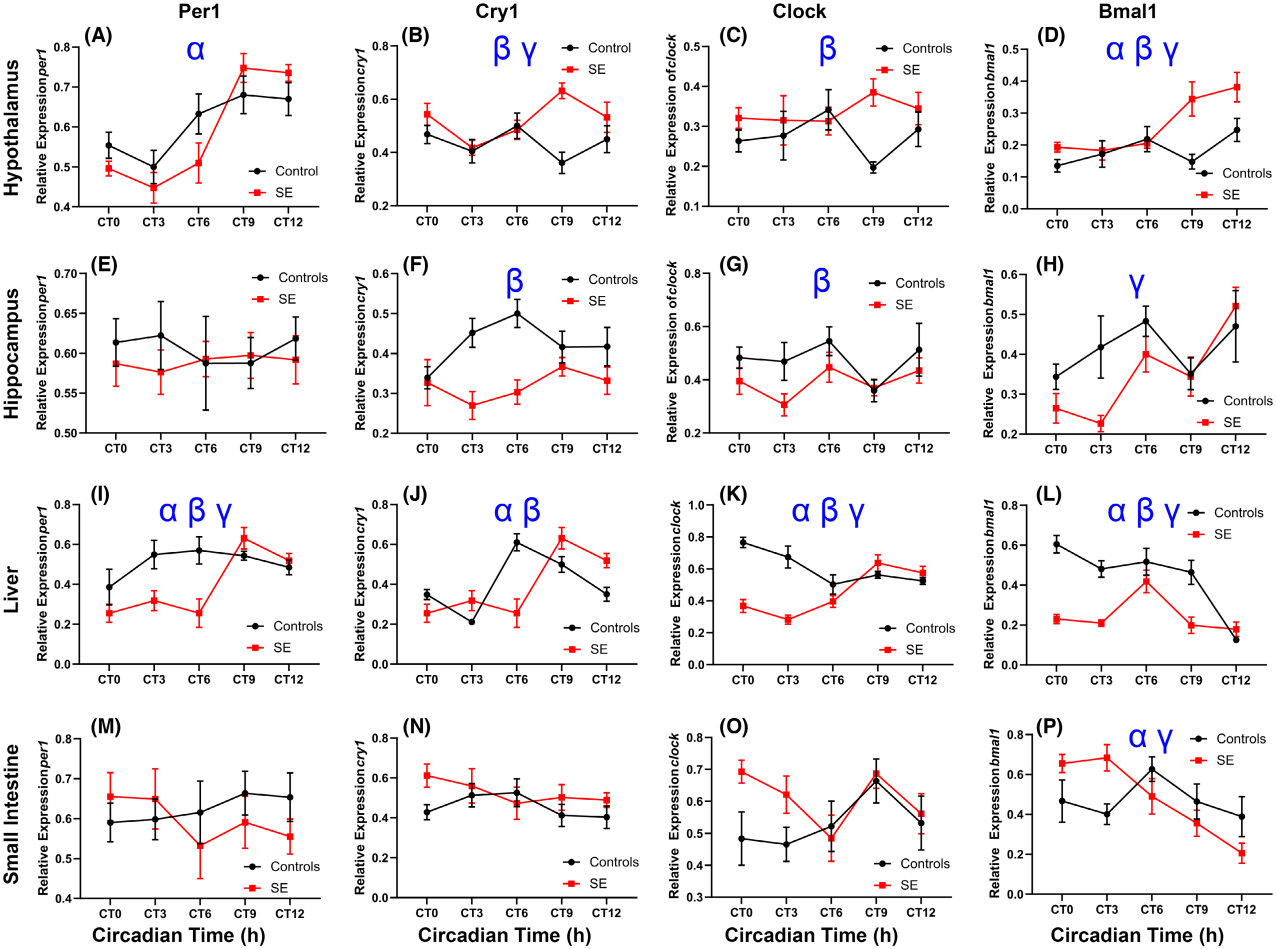
Gene expression for control and kainic acid status epilepticus (KASE) male rats. The hypothalamus is displayed in panels (A–D), the hippocampus is displayed in panels (E–H), the liver is displayed in panels (I–L), and small intestine in panels (M–P). *α* represents a main effect for time; *β* represents a main effect for condition. *γ* represents a condition by time interaction. X‐axis is displayed in circadian time hours where CT0 is defined as the start of the day. The Y‐axis displays the expression of each gene of interest relative to housekeeping genes. KASE (*n* = 8) and shams (*n* = 8) per CT.

*Per1* expression in hypothalamic tissue showed a main effect for time, *F*_(4, 79)_ = 15.269; *P* < 0.01. *Cry1* had a main effect for condition, *F*_(1, 79)_ = 10.42; *P* < 0.01, and a condition by time interaction, *F*_(4, 79)_ = 3.585; *P* < 0.05. *Clock* showed a main effect for epilepsy, *F*_(1, 79)_ = 5.409; *P* < 0.05. *Bmal1* had a main effect for epilepsy, *F*_(1, 79)_ = 13.119; *P* < 0.01, a main effect for time, *F*_(4, 79)_ = 6.381; *P* < 0.01, and an epilepsy by time interaction, *F*_(4, 79)_ = 3.331; *P* < 0.05. Results for hypothalamic gene expression are displayed in Figure 2A–D.

#### 3.2.2 Hippocampus

*Per1* expression showed no significant differences between groups. *Cry1* had a main effect for epilepsy, *F*_(1, 77)_ = 18.907; *P* < 0.01. *Clock* had a main effect of epilepsy, *F*_(1, 76)_ = 5.703; *P* < 0.05. *Bmal1* showed a main effect for time, *F*_(4, 76)_ = 5.218; *P* < 0.01. Results for hippocampal clock gene expression are displayed in Figure 2E–H.

#### 3.2.3 Liver

Liver *Per1* expression displayed a main effect for epilepsy, *F*_(1, 78)_ = 9.439; *P* < 0.01, a main effect for time, *F*_(4, 78)_ = 6.193; *P* < 0.01, and a epilepsy by time interaction, *F*_(4, 78)_ = 4.435; *P* < 0.01. *Cry1* expression had a main effect for epilepsy, *F*_(1, 78)_ = 11.804; *P* < 0.01, and time, *F*_(4, 78)_ = 25.169; *P* < 0.01. *Clock* had a main effect for epilepsy, *F*_(1, 78)_ = 32.198; *P* < 0.01, a main effect for time, *F*_(4, 78)_ = 4.191; *P* < 0.01, and an epilepsy by time interaction, *F*_(4, 78)_ = 14.601; *P* < 0.01. *Bmal1* had a main effect for epilepsy, *F*_(1, 78)_ = 49.673; *P* < 0.01, a main effect for time *F*_(4, 78)_ = 15.609; *P* < 0.01, and an epilepsy by time interaction, *F*_(4, 78)_ = 7.802; *P* < 0.01. Liver expression is displayed in Figure 2I–L.

#### 3.2.4 Small intestine

We found no significant differences in *Per1*, *Cry1*, or *Clock* expression in small intestine tissue (*P* > 0.05). *Bmal1* showed a main effect of time, *F*_(4, 78)_ = 4.893; *P* < 0.01, and a epilepsy by time interaction, *F*_(4, 78)_ = 4.893 *P* < 0.01. Small intestine core circadian clock gene expression is displayed in Figure 2M–P.

## 4 DISCUSSION

Our results demonstrate that the core circadian clock genes are differentially altered in two distinct rat models of chronic epilepsy. The rats with absence epilepsy (ie, GAERS) showed diurnal dysregulation of all core circadian clock genes, *Per1*, *Cry1*, *Clock*, and *Bmal1*, when compared to NECs, or had an epilepsy by time interaction where groups significantly varied depending on the time of day. This suggests that the circadian system is severely desynchronized in GAERS. Conversely, we observed a more modest diurnal dysregulation of clock gene expression in KASE rats. Nevertheless, this gene dysregulation was more pronounced in *Cry1*, *Clock*, and *Bmal1* expression in the hypothalamus and liver, and *Cry1* and *Clock* in the hippocampus when KASE rats were compared with shams.

Within the hypothalamus of both NEC and GAERS rats, *Per1* and *Cry1* showed decreased expression, but *Clock* and *Bmal1* showed increased expression. Since these form heterodimers and the *Per/Cry* heterodimer is the negative regulator of the clock in that it suppresses the activity of *Clock* and *Bmal1*, it is possible that this decreased expression could change the period of the 24‐h feedback loop.^31^, ^32^ Given that the human circadian clock runs close to 24 h, with a period estimated to be about 24.2 h,^33^ further studies would be needed to determine whether the GAERS epileptic hypothalamus had been significantly changed from 24 h and, if so, the repercussions for circadian rhythms in GAERS seizure expression. The hippocampus showed increases in *Per1*, *Cry1*, and *Bmal1* expression with a decrease in *Clock*. While the hypothalamus and hippocampus are not the primary seizure‐generating regions in the GAERS,^19^ it is interesting to see the dysregulation of genes in these regions, suggesting brain‐wide circadian gene dysregulation. Altered clock gene expression has not been studied in epilepsy comorbidities. However, *per* gene polymorphisms correlate with increased depression risk and anxiety disorders.^34^, ^35^ Similarly, altered hypothalamic and hippocampal *Per1* and *Bmal1* expression patterns have been described in a murine model of depressive‐like behavior.^36^, ^37^ Currently, it is unclear the involvement of clock genes in the formation of memory; however, reduction in hippocampal *Per1* using short interfering RNA, impairs learning and *Per1* knockouts show perturbed hippocampal learning‐dependent histone modifications.^38^, ^39^ Given that epilepsy patients report difficulties with memory formation and comorbid affective disorders^40^, ^41^, ^42^ in addition to the disturbed clock genes we found in the current study, it is possible that disrupted circadian function could be interfering with hippocampal‐based learning and memory. Within GAERS liver, *Per1* expression showed a marked increase, while *Cry1* and *Clock* exhibited flattened diurnal expression, and *Bmal1* exhibited altered diurnal expression. Interestingly, mice exposed to toxins including capsaicin and acetaminophen show elevated levels of *Per1* and those lacking *Per1* showed reduced toxin clearance.^43^ While there is much research on ASMs and hepatic toxicity,^44^ there is little information on liver function prior to the administration of ASMs. The GAERS rats may have increased hepatic load prior to drug administration which may serve as an important biomarker for this condition. We also found overexpression of genes in the small intestine. While the connection between epilepsy and the enteric nervous system is well established, it is unclear whether the disease state modifies enteric function, or poor enteric function contributes to the epileptic disease state.^45^

In the KASE rats, *Cry1* and *Clock were* dysregulated in the hippocampus compared with controls. While the role of clock genes in the hippocampus is not well defined, they may play a role in excitability rhythms.^46^, ^47^ We saw no specific effects of epileptic condition for *Per1* in the hypothalamus, hippocampus, or small intestine, and *Cry1* and *Clock* in the small intestine. However, what caught our attention was the lowered expression of circadian clock genes in the liver and the lack of significant changes in the small intestine. There is strong evidence that repeated perturbation of liver circadian function can lead to metabolic disruption^48^ and reports of altered systemic metabolic profiles in KASE rats, which persist even after ASM treatment.^25^ There are some data suggesting lowered clock gene expression is associated with increased epileptogenesis^49^ and some evidence indicating reduced clock gene rhythms in the pilocarpine‐induced post‐SE model of TLE.^50^ Importantly, genetic deletion of the *Bmal1* gene reduced the electrically induced seizure threshold in knockout mice compared with wild‐type mice, suggesting that *Bmal1* contributes to epileptic excitability.^51^ In addition, evidence has shown reduced *Clock* gene expression in the brain of drug‐resistant epilepsy patients, and that targeted deletions of the *Clock* gene in excitatory and parvalbumin‐expressing inhibitory neurons of transgenic mice results in decreased thresholds of pentylenetetrazol‐induced seizures, increased epileptiform discharges, and seizures arising from sleep.^52^ Furthermore, dysregulated *Clock*/*Bmal1* transcription factors might contribute to epileptogenesis, potentially modifying gene expression clusters related to synaptic function, mitochondrial function, and ATPase activity.^52^, ^53^ Importantly, these pathways have been described previously in different single‐omics and multi‐omics assessments in animal models and people with epilepsy.^54^

## 5 CONCLUSIONS, LIMITATIONS, AND FUTURE DIRECTIONS

Studies are necessary to understand the role of the circadian clock genes in the pathogenesis of epilepsy and the development of epilepsy‐related behavioral comorbidities. Understanding, how to manipulate the circadian system may provide a new avenue for targeted development of new epilepsy treatments. Our study would have benefitted from including female rats and characterizing the nocturnal expression of these genes. Future work should also incorporate post hoc analyses of the circadian gene expression across tissues, time, and condition to possibly reveal specific differences that can direct future investigations. Similarly, targeted circadian clock gene analysis of key epileptogenic regions like the thalamocortical circuitry in GAERS, hippocampus CA1 and CA3, and amygdala from KASE rats should be explored next. Lastly, future work should correlate seizure burden and circadian gene expression.

The findings from our current work provide important insight into a new, under‐researched area in understanding and treating different epileptic states. These results contribute to a relatively unexplored field showing that circadian dysfunction is a key outcome of epilepsy. It remains to be seen whether peripheral and central desynchronization plays a role in disease outcomes. Furthermore, we can use our circadian system knowledge and develop interventions such as light therapy, other circadian phase resetting, or resynchronization that may improve seizure outcomes. Finally, future studies could characterize whether a thorough assessment of circadian disruption can be used on epilepsy patients to individualize chronotherapeutic practices.

## CONFLICT OF INTEREST STATEMENT

We confirm that we have read the Journal's position on issues involved in ethical publication and affirm that this report is consistent with those guidelines. The authors report no competing interests related to the current work.

## Data Availability

Data are stored at Monash University servers and can be made available to other researchers by contacting the corresponding authors of this manuscript.
